# Development of Therapeutic Agent for Osteoarthritis via Inhibition of KIAA1199 Activity: Effect of Ipriflavone In Vivo

**DOI:** 10.3390/ijms241512422

**Published:** 2023-08-04

**Authors:** Jiarui Zhang, Yoshihiro Nishida, Hiroshi Koike, Lisheng Zhuo, Kan Ito, Kunihiro Ikuta, Tomohisa Sakai, Shiro Imagama

**Affiliations:** 1 Department of Orthopaedic Surgery, Nagoya University Graduate School of Medicine, Nagoya 466-8560, Japan; zhangjiarui1993@foxmail.com (J.Z.); hiroshikoike@med.nagoya-u.ac.jp (H.K.); ito.kan@med.nagoya-u.ac.jp (K.I.); k-ikuta@med.nagoya-u.ac.jp (K.I.); tosakai@med.nagoya-u.ac.jp (T.S.); imagama@med.nagoya-u.ac.jp (S.I.); 2 Department of Rehabilitation Medicine, Nagoya University Hospital, Nagoya 466-8560, Japan; 3 Rare Cancer Center, Nagoya University Hospital, Nagoya 466-8560, Japan

**Keywords:** extracellular matrix, hyaluronan, ipriflavone, osteoarthritis

## Abstract

This study aimed to clarify the effects of ipriflavone, which effectively reduces KIAA1199 activity, on osteoarthritis (OA) development and progression in an in vivo OA mouse model. The OA model mice were divided into the ipriflavone (200 mg/kg/day) group and the control group. OA onset and progression were evaluated with the Mankin score, and KIAA1199 expression and hyaluronan (HA) accumulation were analyzed by immunostaining. The molecular weight of HA in the cartilage tissue and serum HA concentration were analyzed by chromatography and competitive HA enzyme-linked immunoassay. The effects of ipriflavone on the bovine cartilage explant culture under the influence of IL-1β were also investigated. In the ipriflavone group, Safranin-O stainability was well-preserved, resulting in significant reduction of the Mankin score (*p* = 0.027). KIAA1199 staining positivity decreased and HA stainability was preserved in the ipriflavone group. The serum HA concentration decreased, and the molecular weight of HA in the cartilage tissue increased in the ipriflavone group. The results of the cartilage explant culture indicated that ipriflavone could reduce GAG losses and increase the molecular weight of HA. Thus, ipriflavone may have an inhibitory effect on OA development/progression. Ipriflavone could be a therapeutic drug for OA by targeting KIAA1199 activity.

## 1. Introduction

Osteoarthritis (OA) is a common joint degenerative disease in the aging population, and the prevalence of OA is approximately 30% in those aged ≥ 60 years [1]. The main characteristic symptoms of OA are swelling, inflexibility, pain, and stiffness of the involved joints. The risk factors for OA development include age, sex, injury, mechanical stimulation, and genetic background [2]. Pathological changes in OA include synovial inflammation, osteophyte formation, progressive articular cartilage destruction, and subchondral bone thickening [3].

Hyaluronan (HA) is a polymer of disaccharides, which are composed of D-glucuronic acid and N-acetyl-D-glucosamine, linked via alternating β-(1→4) and β-(1→3) glycosidic bonds. HA is an important component of the cartilage extracellular matrix (ECM), and it binds to aggrecan to maintain the compressive resilience of the cartilage structure [4]. In the synovial fluid, HA maintains joint lubricity, and is involved in the retention of water content in joint cavities. The physical properties of HA in the synovial fluid and cartilage could be changed in the presence of OA. The viscosity and elasticity of the synovial fluid in OA decreases because of the reduced polymerization of HA [5]. Regarding the molecular weight of HA, previous studies have found that high-molecular-weight HA (>1000 kDa) has anti-inflammatory and cartilage protective effects [6,7], whereas low-molecular-weight HA (<200 kDa) could trigger an inflammatory response [8,9,10]. Based on research on the special properties of HA, intra-articular injection of high-molecular-weight HA is commonly used to treat OA, particularly in the knee joints.

There are three HA synthase (HAS) isoforms, namely, HAS1, HAS2, and HAS3. HAS2 plays a major role in the synthesis of high-molecular-weight HA in the articular cartilage. HAS1 and HAS3 are responsible for the synthesis of relatively low-molecular-weight HA and are reported to be very slightly expressed in the articular cartilage [11]. In the OA cartilage, the expression of HAS2 was found to be upregulated [12]. Forced expression of HAS2 in chondrocytes could inhibit the expression of matrix metalloproteinase (MMP)3 and MMP13 and increase aggrecan retention, suggesting HAS2 as one of the potential targets for OA treatment [13,14].

HA-degrading enzymes mainly include HYAL1 and 2, and KIAA1199, also known as CEMIP (cell migration-inducing hyaluronan-binding protein). HYAL1 and 2 require CD44 to induce hyaluronidase activity [15]. KIAA1199 was originally identified as a deafness gene and, subsequently, was found to play crucial roles in HA-degrading activities in chondrocytes, independent from HYAL1 and 2 and CD44. The official name of the gene is CEMIP, cell migration-inducing hyaluronidase 1, also known as CCSP1, HYBID, CEMIP1, TMEM2L, and KIAA1199 (Gene ID: 57214). KIAA1199 was also found to be upregulated in HA-depleted areas in the articular cartilage [16,17]. KIAA1199 deletion (KIAA-knockout mouse) could attenuate OA and increase the molecular weight of HA in the joint tissue in a mouse model of OA with destabilization of the medial meniscus (DMM) [18]. This indicates the importance of KIAA1199 in OA development/progression.

Given the possible crucial roles of KIAA1199 in OA pathogenesis, targeting KIAA1199 to develop a novel OA treatment could be promising. Recently, we discovered that ipriflavone can effectively suppress KIAA1199 activity in a drug-screening approach. Ipriflavone (7-isopropoxyisoflavone) is a synthetic, non-hormonal isoflavone derivative currently used to prevent and treat osteoporosis. The overexpression of the *KIAA1199* gene in rat chondrosarcoma (RCS) cells, which are originally abundant in the HA-rich pericellular matrix, decreases the formation of the HA-rich pericellular matrix. Ipriflavone recovered the pericellular matrix formation of KIAA1199-overexpressed RCS cells. In a collagen-induced arthritis model, ipriflavone significantly improved the symptoms and reduced serum HA concentrations, suggesting the effectiveness of ipriflavone for arthritis, such as rheumatoid arthritis [19]. However, the effects of ipriflavone on OA, which is the most common degenerative disease of the joint, have not been examined yet. In this study, using the DMM mouse model for OA, we investigated the inhibitory effects of ipriflavone on OA development and progression. In addition, to verify the effects of ipriflavone on cartilage tissues under inflammatory conditions, an explant culture of bovine cartilage was subjected to analyses.

## 2. Results

### 2.1. Ipriflavone Inhibited Cartilage Degeneration and GAG Losses in OA Model Mice

**Figure 1:**
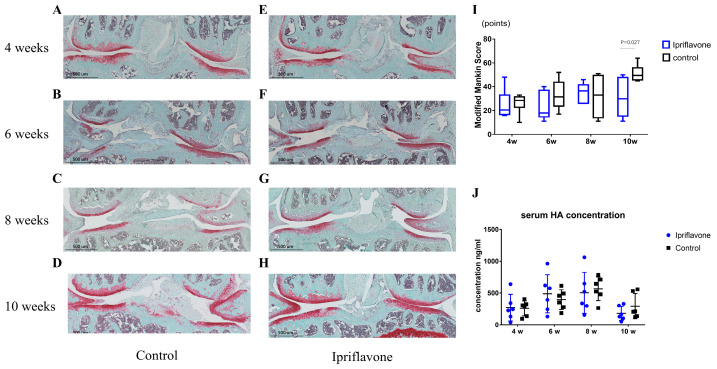
Safranin O staining of mouse knee joints after DMM surgery and serum HA concentrations. Safranin O staining of mouse knee joints in control group (**A**–**D**) and ipriflavone group (**E**–**H**) at 4, 6, 8, and 10 weeks, respectively. (**I**) Modified Mankin score of mouse articular cartilage (*n* = 6 per group); the data show a statistically significant difference at 10 weeks (*p* = 0.027). (**J**) Mouse serum HA concentrations (*n* = 6 per group).

No adverse events, including weight loss, were observed throughout the experimental period in either the ipriflavone group or the control group. Safranin O staining showed that both groups have glycosaminoglycan deposition. The ipriflavone group (Figure 1E,F) had slightly darker staining than the control group (Figure 1A,B) at 4 and 6 weeks. At 8 weeks, cartilage destruction and glycosaminoglycan loss started to appear in the control group (Figure 1C), whereas in the ipriflavone group, the cartilage only experienced partial glycosaminoglycan loss (Figure 1G). At 10 weeks, the cartilage destruction in the control group had become more severe, with erosion occurring under the tidemark (Figure 1D), whereas in the ipriflavone group, the cartilage only showed some fibrillations and glycosaminoglycan loss (Figure 1H). The results of the Mankin score indicated that ipriflavone could reduce the Mankin score and show a statistical difference at 10 weeks (Figure 1I, *p* = 0.027). The results of serum HA concentrations revealed no statistical difference between the ipriflavone and control groups (Figure 1J).

### 2.2. Ipriflavone Decreased KIAA1199 Expression and Increased the Positive Area of HA-Binding Protein (HABP) Staining in the Cartilage

**Figure 2:**
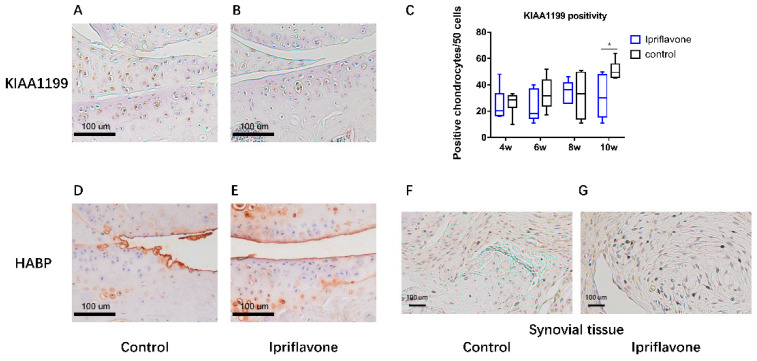
Immunohistochemical staining of KIAA1199 and HABP in mouse knee joints 10 weeks after DMM surgery. KIAA1199 staining of articular cartilage of control (**A**) and ipriflavone groups (**B**) and HABP staining of articular cartilage of control (**D**) and ipriflavone groups (**E**). (**C**) The number of KIAA1199-positive cells in every 50 chondrocytes in medial tibia plateau cartilage. * *p* < 0.05. KIAA1199 staining for synovial tissues in control (**F**) and ipriflavone (**G**) groups.

KIAA1199 positivity in the chondrocytes and HABP in the cartilage tissue were evaluated at the medial tibial plateau, which is the weight-bearing area in the joints. The number of chondrocytes positive for KIAA1199 decreased in the cartilage of the ipriflavone group (Figure 2B) compared with that of the control group (Figure 2A), and a statistically significant difference was found at 10 weeks (Figure 2C). The results of HABP staining showed that the ipriflavone group (Figure 2E) had a larger HABP-positive area than the control group (Figure 2D). Decreased KIAA1199 positivity in the synovial tissues was also observed in the ipriflavone group (Figure 2G) compared to that of control group (Figure 2F) at 10 weeks.

### 2.3. Ipriflavone Could Increase the Molecular Weight of HA in the OA Cartilage

**Figure 3:**
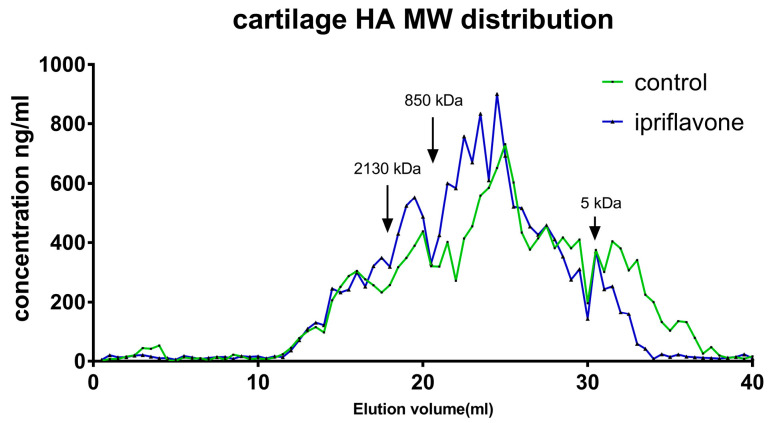
The molecular weight distribution of hyaluronan. HA was extracted from bilateral knee articular cartilage from 6 mice at 10 weeks after DMM surgery. The blue curve represents the ipriflavone group and the green curve represents the control group.

Knee cartilage from six mice was obtained after careful removal of soft tissues under the microscope, and HA was extracted. The results of chromatography showed that the HA remained at a high molecular weight in the ipriflavone group, whereas the HA in the control group shifted to a low molecular weight (Figure 3).

### 2.4. Ipriflavone Reduced the Loss of GAGs under IL-1β Stimulation

**Figure 4:**
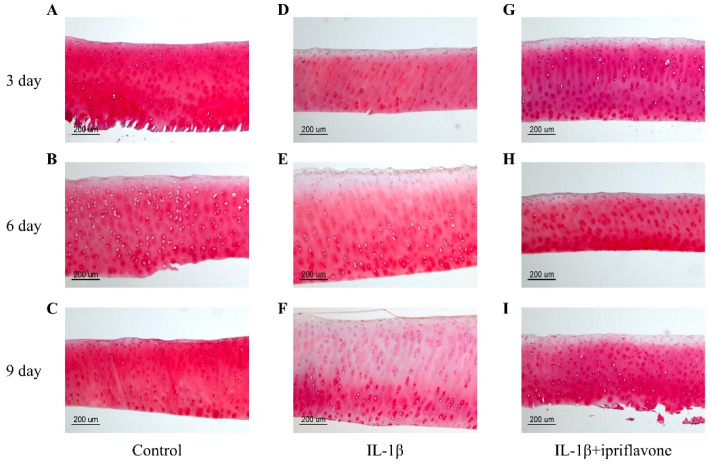
Safranin O staining of bovine cartilage explants. Safranin O staining of bovine cartilage explants in the control group after 3, 6, and 9 days of culture ((**A**), (**B**), and (**C**), respectively), IL-1β group after 3, 6, and 9 days of culture ((**D**), (**E**), and (**F**), respectively), and the IL-1β plus ipriflavone group after 3, 6, and 9 days of culture ((**G**), (**H**), and (**I**), respectively).

The results of Safranin O staining indicated that, compared with control (Figure 4A–C), the staining decreased in a time-dependent manner in the IL-1β group (Figure 4D–F). The time-dependent loss of glycosaminoglycans due to IL-1β was inhibited by ipriflavone (Figure 4G–I).

### 2.5. Ipriflavone Maintained the Deposition and Molecular Weight of HA in IL-1β-Stimulated Cartilage Explants

**Figure 5:**
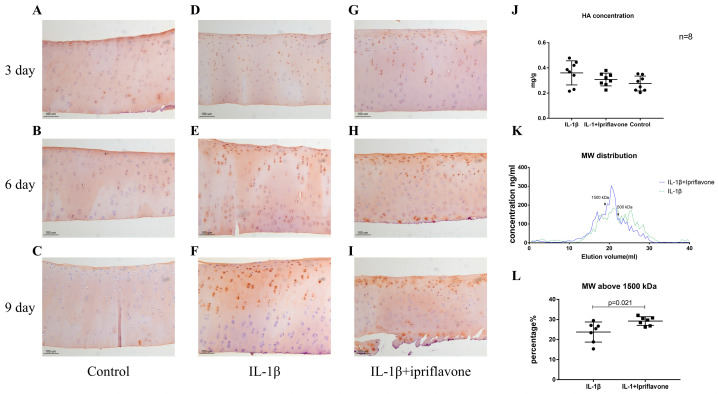
The HABP staining of bovine cartilage explants. Control group after 3, 6, and 9 days of culture ((**A**), (**B**), and (**C**), respectively). IL-1β group after 3, 6, and 9 days of culture ((**D**), (**E**), and (**F**), respectively). IL-1β plus ipriflavone group after 3, 6, and 9 days of culture ((**G**), (**H**), and (**I**), respectively). (**J**) The concentration of HA in bovine cartilage in the 3 groups is depicted (*n* = 8). (**K**) The molecular weight distribution of HA extracted from bovine cartilage in IL-1β and IL-1β plus ipriflavone groups. (**L**) Proportion of high-molecular-weight HA in bovine cartilage from IL-1β and IL-1β plus ipriflavone groups (*n* = 7, *p* = 0.021).

The results of HABP staining showed that the HA positivity had not changed much in the ECM area of the cartilage but decreased in a time-dependent manner in the chondrocytes of the control group (Figure 5A–C). ECM positivity decreased in a time-dependent manner, whereas that in chondrocytes was upregulated by IL-1β (Figure 5D–F). In the ipriflavone group, positivity in the ECM was maintained, whereas it increased in the chondrocytes (Figure 5G–I) compared with the IL-1β and control groups. In the cartilage, the HA content in the IL-1β group was high compared with that in the control and ipriflavone groups (Figure 5J). The results of chromatography showed that the curve of the ipriflavone group shifted to the left, which suggests the maintenance of high-molecular-weight HA (Figure 5K). We calculated the portion of high-molecular-weight HA (>1500 kDa) and found that the ipriflavone group had significantly higher proportions of high-molecular-weight HA than the IL-1β group (Figure 5F, *p* = 0.021). Because the calibration range was too narrow, the average molecular weight of HA in the two groups could not be calculated. However, since the average molecular weight of the polymer is largely determined by the high-molecular-weight portion [20], ipriflavone might maintain a high average HA molecular weight compared with IL-1β.

### 2.6. Immunohistochemical Staining for KIAA1199 in Bovine Cartilage Explants

**Figure 6:**
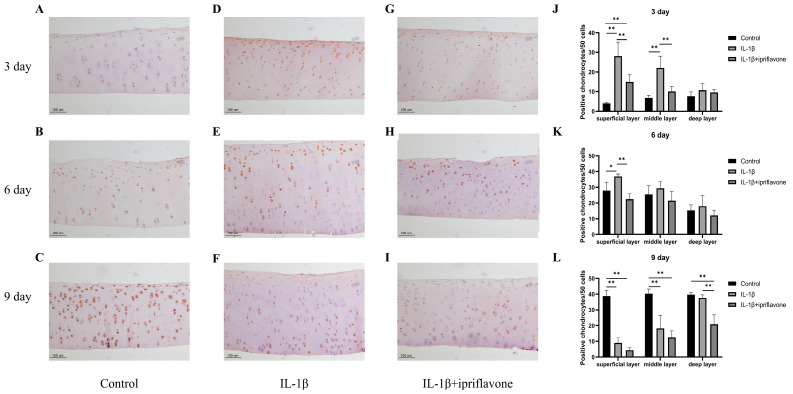
Immunohistochemical (IHC) staining of KIAA1199 in bovine cartilage explants. IHC staining of KIAA1199 in control group after 3, 6, and 9 days of culture ((**A**), (**B**), and (**C**), respectively). IHC staining of KIAA1199 in IL-1β group after 3, 6, and 9 days of culture ((**D**), (**E**), and (**F**), respectively). IHC staining of KIAA1199 in IL-1β plus ipriflavone group after 3, 6, and 9 days of culture ((**G**), (**H**), and (**I**), respectively). * *p* < 0.05, ** *p* < 0.01. The number of KIAA1199 positive cell in every 50 chondrocytes in superficial, middle, deep layer of bovine cartilage after 3 (*n* = 4 per group), 6 (*n* = 4 per group) and 9 (*n* = 5 per group) days of culture ((**J**), (**K**) and (**L**), respectively). * *p <* 0.05. ** *p <* 0.01.

The results of the 3-day culture showed that KIAA1199 positivity was higher in the IL-1β group (Figure 6D) than in the control group (Figure 6A), whereas that in the ipriflavone group (Figure 6G) was lower than that of the IL-1β group (Figure 6D), and KIAA1199 was mostly expressed in the superficial layer of the cartilage in both groups, whereas the control group expressed only small amounts (Figure 6A). The results of the 6-day culture showed that KIAA1199 expression increased in both the IL-1β and ipriflavone groups, spreading to the middle layer of the cartilage (Figure 6E,H). The control group (Figure 6B) was also positive for KIAA1199. The results of the 9-day culture showed that KIAA1199 was expressed mainly in the middle and deep layers of the cartilage in the IL-1β (Figure 6F) and ipriflavone (Figure 6I) groups, whereas it was highly expressed in every layer of cartilage in the control group (Figure 6C). The KIAA1199-positive cell ratio in each (superficial, middle, and deep) layer at the 3-day, 6-day, and 9-day time points were calculated and graphed in Figure 6J, K, and L, respectively.

### 2.7. mRNA Expression of KIAA1199 and Related Genes

**Figure 7:**
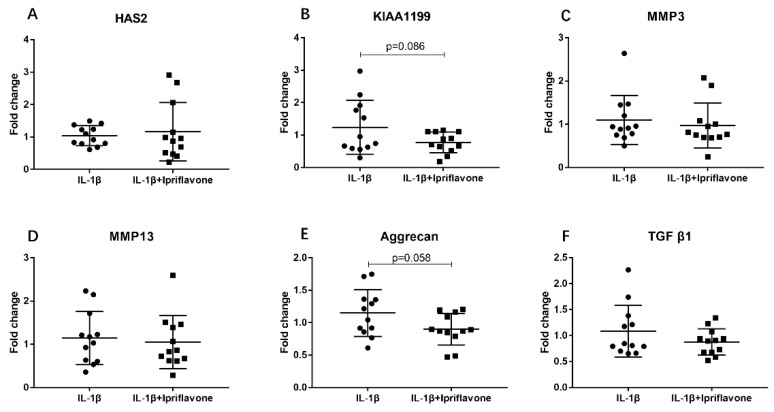
mRNA expression of ECM-related genes in bovine cartilage explants. mRNA expression of *HAS2* (**A**), *KIAA1199* (**B**), *MMP3* (**C**), *MMP13* (**D**), *Aggrecan* (**E**), and *TGF β1* (**F**) in bovine cartilage 1 day after treatment with IL-1β or IL-1β plus ipriflavone, determined by real-time PCR amplification (*n* = 12, respectively). The data presented are the average ± S.D. of relative mRNA expression values standardized by GAPDH mRNA expression.

No significant difference was observed between the IL-1 group and the ipriflavone group in the mRNA expression levels of *HAS2*, *MMP13*, *MMP3*, *aggrecan*, and *TGF-β1* (Figure 7A–F). Regarding *KIAA1199*, the expression of mRNA tended to be low in the ipriflavone group (Figure 7B, *p* = 0.086). The animal experiments showed no statistically significant difference between the control group and the ipriflavone group in ECM-related genes in the knee joint articular cartilage (Supplement Figure S1).

## 3. Discussion

The results of this study revealed that ipriflavone, which was shown to inhibit KIAA1199 [19], could inhibit cartilage degradation in a mouse OA model with DMM surgery. In addition, the inhibitory effects of ipriflavone were confirmed by the results of the bovine cartilage explant culture under IL-1β stimulation. Since no drugs have been developed that can change the pathology of OA, the results of this study indicate that ipriflavone could be a candidate anti-OA drug.

HA is the crucial component of the cartilage ECM and plays pivotal roles in the maintenance of the ECM by forming a proteoglycan aggregate in the ECM. HA is synthesized by HAS in chondrocytes. There are three HAS isoforms, namely, HAS1, HAS2, and HAS3. Among them, HAS2 is the main HAS in chondrocytes and is responsible for producing high-molecular-weight HA in the cartilage [21]. The balance between HA depolymerization and synthesis in OA is unstable depending on the stage [12,17]. The unbalanced metabolic activity of HA leads to HA loss in the OA cartilage while increasing the total amount of HA in the synovial fluid and decreasing its molecular weight [22,23]. High-molecular-weight HA was found to have anti-inflammatory effects and reduce glycosaminoglycan loss in the OA cartilage, and intra-articular injections of high-molecular-weight HA are widely used for OA treatment [6,7]. Studies have also tried to treat OA by increasing endogenous HA synthesis [24,25]. In the present study, ipriflavone did not increase HAS2 mRNA expression in bovine explant cultures, suggesting that the inhibitory effects of ipriflavone may not be mediated by alterations of HAS2 expression. On the contrary, few studies aimed to suppress the catabolic activity of HA to inhibit OA progression.

KIAA1199 was originally discovered as a deafness gene and was subsequently found to play a central role in degrading high-molecular-weight HA in the chondrocytes through rapid vesicle endocytosis and recycling, whereas HYAL1/2 and CD44 knockdown could not alter the ability of chondrocytes to degrade high-molecular-weight HA [17]. The results of studies analyzing KIAA1199’s involvement in OA indicated that KIAA1199 was highly expressed in HA-depleted areas in OA cartilage and highly correlated with cartilage destruction [16,26]. In KIAA1199-knockout mice, cartilage degeneration was relieved, and the molecular weight of HA in the knee joint tissue increased [18]. These previous studies have indicated that the suppression of HA-degrading activity could attenuate cartilage degeneration and OA development. However, since these experiments attempted to suppress KIAA1199 by genetic manipulation, it is difficult to implement in clinical settings. Ipriflavone, which has HA-degrading activity, has low toxicity and was discovered using a drug-repositioning technique. In this study, ipriflavone showed an inhibitory effect on OA progression in an in vivo mouse OA model, suggesting that this drug may be used in clinical practice.

In a previous study, ipriflavone showed the ability to suppress KIAA1199 activity and inhibited HA depolymerization in fibroblast-like synoviocytes (FLSs) stimulated with TNF-α, whereas in the present study, ipriflavone reduced the protein expression of KIAA1199 in the OA cartilage [19]. The mechanism by which ipriflavone directly suppresses KIAA1199 protein expression is unknown. KIAA1199 expression is differentially affected by cell type or cytokine stimulation. In human skin fibroblasts, TNF-α could upregulate KIAA1199 expression, whereas IL-1β could downregulate it. A mixture of cytokines TNF-α, IL-1β, and IL-6 could downregulate KIAA1199 expression [27]. Meanwhile, in OUMS-27 cells (a human chondrosarcoma cell line), both TNF-α, IL-1β, and IL-6 could upregulate KIAA1199 expression [28]. IL-1β induces KIAA1199 expression and migration in pancreatic ductal adenocarcinoma cells [29]. These studies have highlighted the complexity of the KIAA1199 signaling pathway in different organs. Ipriflavone may have different effects on KIAA1199 in FLSs and chondrocytes, which should be investigated further.

The aggrecan core protein interacts with HA to form large complexes that provide the cartilage with a hydrated structure that furnishes the cartilage with its compressive stiffness and load-bearing properties. Aggrecan was depleted in the OA cartilage, and the MMP and ADAMTS (a disintegrin and metalloproteinase with thrombospondin motifs) families were important enzymes for aggrecan degradation [30]. The expression level of the aggrecan gene is upregulated in early-stage OA cartilage and downregulated in the late stage [31,32]. Previous studies have indicated that aggrecan expression was related to the severity of cartilage degradation. The results of the present study depicted that Safranin O staining was preserved by treatment with ipriflavone in vivo and in bovine explant culture, possibly via the suppression of the HA-degrading activity of ipriflavone, leading to the maintenance of proteoglycan aggregates in the ECM. In the explant cultures, KIAA1199 staining was upregulated at the 9-day time point. Since the explant culture does not reflect the physiological conditions for articular cartilage, the high expression of KIAA1199 at day 9 in the control could be due to the longer culture period. Safranin O staining, which reflects proteoglycan deposition, did not decrease in control articular cartilage in the 9-day culture. The Safranin O staining positivity does not decrease until a certain amount of proteoglycan is lost. Therefore, even if the expression of KIAA1199 increased after 9 days in the control culture, Safranin O staining can still be considered to be preserved at this time point.

In previous research, ipriflavone has shown the ability to downregulate the serum HA concentration and HA deposition in the synovium in a collagen-induced mouse model [19]. In the present study, ipriflavone could not downregulate the serum HA concentration in the DMM model mice. Thus, the differing pathological mechanisms between OA and RA contribute to the different results. In RA, FLSs in the synovial membrane play a considerably important role in RA pathogenesis. When activated by the auto-immune system or inflammation, FLSs start to secret inflammatory cytokines and enzymes including KIAA1199, which leads to cartilage destruction and release of ECM components. The released matrix products lead to the activation of FLSs to a stable tumor-like phenotype, making the cartilage destruction permanent. In OA, external stress (injury, mechanical load, and inflammation) first act on the chondrocytes, which leads to the loss of phenotypic stability and expression of matrix-degrading molecules (ADMATS, MMP, and KIAA1199), resulting in the degradation of the cartilage ECM [33]. Clinical studies have shown that the increase in serum HA levels is higher in RA than in OA [34,35]. In previous studies, serum HA concentrations in CIA mice was approximately 1000–3000 ng/mL; however, in the present experiment, the serum HA concentrations in the DMM model mice was approximately 500 ng/mL [19,36]. Although synovial tissue inflammation is common in OA and RA, the severity and prevalence are different. OA synovial tissue displays a mild-to-moderate degree of inflammation, whereas in RA, the inflammation is moderate to severe. The severity of synovitis in OA is much lower than that in RA [36,37,38]. Increased deposition of HA was found in the arthritic synovium tissue [22,39]. Although OA and RA have different disease mechanisms, ipriflavone, which has an inhibitory effect on HA degradation, was able to suppress disease progression in both disorders.

In the bovine cartilage explant experiment, ipriflavone maintained high-molecular-weight HA under IL-1β stimulation; however, the total content of HA in the ipriflavone and control groups appeared to be less than that in the IL-1β group. This finding is inconsistent with those of previous clinical studies. Previous studies reported that the HA content in OA cartilage decreased [40,41]. HA synthesis in chondrocytes could be activated by IL-1, as the mRNA expression of HAS2 and 3 was upregulated [12,42]. The increased production of HA was found to be either released into the culture medium or internalized in the chondrocyte cytoplasm [25,43]. Another study showed that, under IL-1α stimulation, the newly synthesized HA will move out from the cell-associated matrix to reside in the further removed matrix, which represents the interterritorial matrix in the cartilage, and the HA content in the further removed matrix increased significantly [44]. Therefore, the high content of HA in the IL-1β group may be due to that the culture period that was too short for HA to be released.

Ipriflavone was initially considered a promising drug for the treatment of osteoporosis; however, subsequent experiments proved that ipriflavone could not stop bone loss [45]. Recently, ipriflavone was proven to have the ability to suppress the HA degradation activity dominated by KIAA1199 and increased pericellular matrix formation around RCS cells [19]. A previous study also found that ipriflavone and its metabolites (MET I, III, and V) could significantly enhance glycosaminoglycan and collagen II synthesis in chondrocytes [46]. Other studies have reported that ipriflavone could attenuate cartilage degeneration by blocking the Indian hedgehog pathway [47]. This evidence proved that ipriflavone could be a potential drug for OA through mechanisms other than its inhibition of KIAA1199.

This study has several limitations. First, most of the ipriflavone that is absorbed into the human body will be mainly metabolized into five metabolites; thus, the specific mechanism of ipriflavone and its metabolites in the human body must be clarified. Second, owing to the technical limitations, we did not measure KIAA1199 activity in the cartilage. Third, the effect of ipriflavone on complete OA must be verified. In addition, experiments with different concentrations of ipriflavone in mouse models of OA have not been performed. Different concentrations may have given different results.

## 4. Materials and Methods

### 4.1. Development of OA Mice Model with Destabilization of Medial Meniscus

DMM surgery was performed as previously described [48]. Ten-week-old male C57BL/6J mice were anesthetized with intraperitoneal injection of 0.2 mL of three types of mixed anesthetic agents [49]. The skin on both knee joints was depilated using a depilatory cream. Under sterilized conditions, the right knee skin was incised, followed by medial parapatellar arthrotomy and blunt dissection of the anterior fat pad to expose the anteromedial meniscal tibial ligament, which was then dissected. Sham surgery was performed on the left side knee by making a skin incision and medial parapatellar arthrotomy. The mice were divided into two groups: an ipriflavone group (*n* = 24) and a control group (*n* = 24). Ipriflavone was dissolved in a 0.5% methylcellulose solution. Two weeks after DMM surgery, oral administration of ipriflavone (LKT Labs, St. Paul, MN, USA) at a dose of 200 mg/kg per day was started in the ipriflavone group, and a 0.5% methylcellulose only solution was given to the control group. At 4, 6, 8, and 10 weeks after DMM surgery, 6 mice from each group were sacrificed, and their knee joints were collected for histological analysis. To investigate the changes in the molecular weight of HA and gene expression profiles in vivo, another experiment was performed. Ten-week-old male C57BL/6J mice (*n* = 20) underwent DMM surgery on both knees and were divided into an ipriflavone group (*n* = 10) and control group (*n* = 10). Two weeks after the DMM surgery, the ipriflavone group received 200 mg/kg/day ipriflavone orally, whereas the control group received a 0.5% methylcellulose solution daily. At 10 weeks, the mice were sacrificed, and both knee joints were collected and subjected to PCR (*n* = 4) and cartilage HA molecular-weight (*n* = 6) analyses.

### 4.2. Histological Evaluation of Mouse Knee Joints

The collected knee joints were fixed in 10% formalin, dehydrated, embedded in paraffin, sectioned onto slides, and stained with Safranin O. The modified Mankin score was used to evaluate the level of degradation of the articular cartilage in coronal sections of the knee joints [50]. Serial coronal sections on four quadrants, including medial, and lateral femoral condyles, and medial and lateral tibial plateaus, were examined, and the maximally affected section was evaluated and scored. Grading was scored by two double-blinded observers three times, and the average value was calculated. The interval between each scoring was more than 2 weeks. The scoring criteria included seven aspects: cartilage structure, loss of Safranin O staining, tidemark duplication, fibrocartilage, hypertrophic chondrocytes in the calcified cartilage, subchondral bone changes, and chondrocytes in the uncalcified cartilage.

### 4.3. Immunohistochemistry

To analyze the deposition of HA and KIAA1199 in tissues, paraffin sections of mouse knee joints and bovine cartilage explants were subjected to immunohistochemical analysis. The sections underwent a hydration process and were washed with phosphate-buffered saline (PBS) (pH 7.4) three times and then incubated with chondroitinase ABC solution (0.25 U/mL, pH 8.0; Sigma Aldrich, Saint Louis, MO, USA) for 2 h at 37 °C to unmask the deposition of ECM protein around the chondrocytes. Primary antibodies, rabbit anti-KIAA1199 polyclonal antibody (1:200 dilution; Proteintech, Rosemont, IL, USA) and biotinylated HABP (1:200 dilution; Hokudo, Japan), were incubated with the specimens at 4 °C overnight. After washing with PBS, the sections were incubated with secondary antibody and streptavidin using an SAB-POKit (Nichirei Bio, Tokyo, Japan) to visualize the protein of interest.

### 4.4. Preparation of Cartilage Glycosaminoglycans

HA was extracted from articular cartilage according to a previous report [51]. In this method, deferoxamine was used to inhibit the depolymerization of high-molecular-weight HA during the extraction process using proteases. Cartilage tissues obtained from mouse knee joints or bovine cartilage explants were placed in liquid nitrogen and crushed to powder using a bead crusher (TAITEC Corp., Saitama, Japan). The powder was then immersed in 600 μL of a 0.2 N NaOH solution at room temperature overnight. After neutralization with 1 N HCl, the solution was adjusted to contain 0.01 M CaCl_2_, 0.15 M Tris, 0.15 M NaCl, 5 mM deferoxamine, and 20 U/mL protease E at pH 8 and subjected to incubation at 55 °C for 8 h. Protease E was inactivated by heating the solution up to 95 °C for 15 min, centrifuging the solution at 12,000 rpm for 10 min, and removing the insoluble material. The supernatant was mixed with three volumes of 95% ethanol containing 1.3% potassium acetate, frozen at −20 °C for 30 min, and then centrifuged at 12,000 rpm for 10 min at 4 °C.

### 4.5. Chromatography Analysis of HA Molecular Weight

A Sephacryl S-100 column (0.7 × 40 cm) was used to separate the glycosaminoglycan extract. The column was eluted with PBS at a flow rate of 0.5 mL/min, and fractions were collected every 0.5 mL. The HA concentration, with cartilage glycosaminoglycans in each fraction, was determined three times by competitive ELISA, and the average value was calculated. To calibrate the column, HA with known molecular weights was used (2130, 850, 150, and 5.6 kDa; Seikagaku Corp., Tokyo, Japan).

### 4.6. Serum HA in DMM Model Mice

To determine the serum HA level in DMM mice, blood samples were collected through the posterior vena cava under anesthesia. The blood samples were left at room temperature for half an hour and then centrifuged to obtain the serum from the top layer. The serum HA concentration of each sample was tested three times by competitive ELISA, and the average value was calculated.

### 4.7. Cartilage Explant Cultures

Full-thickness bovine cartilage was collected from the metacarpophalangeal joints of young adult steers (aged 18–24 months), which were purchased from Nagoya City Central Wholesale Market in Japan with institutional approval. Full-thickness bovine cartilage was cultured in low-glucose DMEM medium with 4% FBS for 24 h. The medium was then replaced, and tissues were divided into three groups: IL-1β group, ipriflavone group, and control group. The control group contained 0.1% DMSO, whereas the IL-1β group contained 10 ng/mL IL-1β (R&D Systems, Minneapolis, MN, USA) and 0.1% DMSO. The ipriflavone group contained 10 ng/mL IL-1β, 10 µM ipriflavone, and 0.1% DMSO. The medium was changed every 3 days. Since ipriflavone has poor solubility in water, DMSO was used as the solvent. On day 1 of culture, the treated explants from each group were partially collected for PCR analysis. On days 3, 6, and 9, the cartilage was collected, fixed with 4% buffered paraformaldehyde overnight at 4 °C, rinsed in 30% sucrose/PBS, and embedded in paraffin for histological analysis. A part of the cartilage on day 9 was weighted, and HA was extracted to calculate the HA amount.

### 4.8. Real-Time PCR

Cartilage specimens were placed in liquid nitrogen and crushed into powder with a μt-12 bead crusher (TAITEC Corp.) Total RNA was isolated from the powdered tissue using an RNeasy Mini Kit (Qiagen, Venlo, The Netherlands) and reverse-transcribed to complementary DNA (cDNA) according to the manufacturer’s protocol. The cDNA was subjected to real-time RT-PCR for semi-quantification of messenger RNAs (mRNAs) using a LightCycler480 II (Roche, Basel, Switzerland). The target HA-related genes in mouse knee articular cartilage were *HAS1*, *HAS2*, *HAS3*, *HYAL1*, *HYAL2*, and *KIAA1199*, and the primer sequences are shown in Table 1. The target ECM-related genes in the bovine cartilage were *HAS2*, *KIAA1199*, *MMP13*, *MMP3*, *AGCN*, and *TGF-β1*, and the primer sequences are shown in Table 2. The relative expression level of mRNA of each gene was normalized with that of glyceraldehyde-3-phosphate dehydrogenase.

### 4.9. Statistical Analysis

Data are presented as mean ± SD. Statistical analyses were performed either by the Kruskal–Wallis test or two-tailed *t*-test, followed by Bonferroni corrections. *p*-values less than 0.05 were considered significant. The statistical analyses were performed using SPSS Statistics 27.0 for Microsoft Windows (IBM Corp., Armonk, NY, USA).

## 5. Conclusions

Ipriflavone was found to suppress OA progression in OA model mice. Ipriflavone has already been used in the clinical setting for osteoporosis for a long time, and its safety has been confirmed; thus, it may be a promising drug for OA.

## Figures and Tables

**Table 1:** Sequences of specific primer pairs for each gene (mouse).

HAS1	Forward	5′-TCCTCTGGGTCTATACAGAAACAATC-3′
	Reverse	5′-CGGTTGGTGAGGTGCCTGT-3′
HAS2	Forward	5′-GATTATGTACAGGTGTGTGAC-3′
	Reverse	5′-CCTCTAAGACCTTCACCATC-3′
HAS3	Forward	5′-GATGTCCAAATCCTCAACAAG-3′
	Reverse	5′-CAAAGGCCCACTAATACATTG-3′
HYAL1	Forward	5′CAAGTACCAAGGAATCATGCCAG-3′
	Reverse	5′-GCGGACACAGCGACCATG-3′
HYAL2	Forward	5′-TGTGGCTCTCACCTGGACCTTATGA-3′
	Reverse	5′-AGATGGTATGGGTGCTCTGCTAAG-3′
KIAA1199	Forward	5′-ATATACAGGCCACAACAATG-3′
	Reverse	5′-AAGCAAACCTGTAATCTTGG-3′
GAPDH	Forward	5′-ACCCAGAAGACTGTGGATGG-3′
	Reverse	5′-CACATTGGGGGTAGGAACAC-3′

**Table 2:** Sequences of specific primer pairs for each gene (bovine).

HAS2	Forward	5′-CGAAGTGTGGATTATGTACAGGTTT-3′
	Reverse	5′-GACACCTCCAACCATGGGAT-3′
KIAA1199	Forward	5′-CATGCTGGCATGGCCTCT-3′
	Reverse	5′-ACTCAGCTGACCCTGTGGAT-3′
MMP3	Forward	5′-CACCATGGAGCTTGTTCAGCAATA-3′
	Reverse	5′-GTCACCTCCAACCCCAGAAA-3′
MMP13	Forward	5′-CCAGTTTGCAGAGAGCTACC-3′
	Reverse	5′-CTGCCAGTCACCTCTAAGCC-3′
AGCN	Forward	5′-AAGAGAGCCAAACAGCCGAC-3′
	Reverse	5′-TCGCACAGCTTCTGGTCTGT-3′
TGF-β 1	Forward	5′-ATACACAGTACAGCAAGGTCCTG-3′
	Reverse	5′-ACGTAGTACACGATGGGCAG-3′
GAPDH	Forward	5′-AAGGTCGGAGTGAACGGATTC-3′
	Reverse	5′-ATGGCGACGATGTCCACTTT-3′

## Data Availability

Most data is contained within the article. Other data will be available on request within 6 months of this publication via direct communication with the corresponding author.

## Supplementary Materials

The supporting information can be downloaded at: https://www.mdpi.com/article/10.3390/ijms241512422/s1.
