# Supplementary material for: Development of Therapeutic Agent for Osteoarthritis via Inhibition of KIAA1199 Activity: Effect of Ipriflavone In Vivo
Source: Int J Mol Sci. 2023 Aug 4;24(15):12422. doi: 10.3390/ijms241512422 (PMC10419624; doi:10.3390/ijms241512422)
Supplement: Supplementary file 1 [file ijms-24-12422-s001.zip › ijms-2523120-supplementary.pdf]

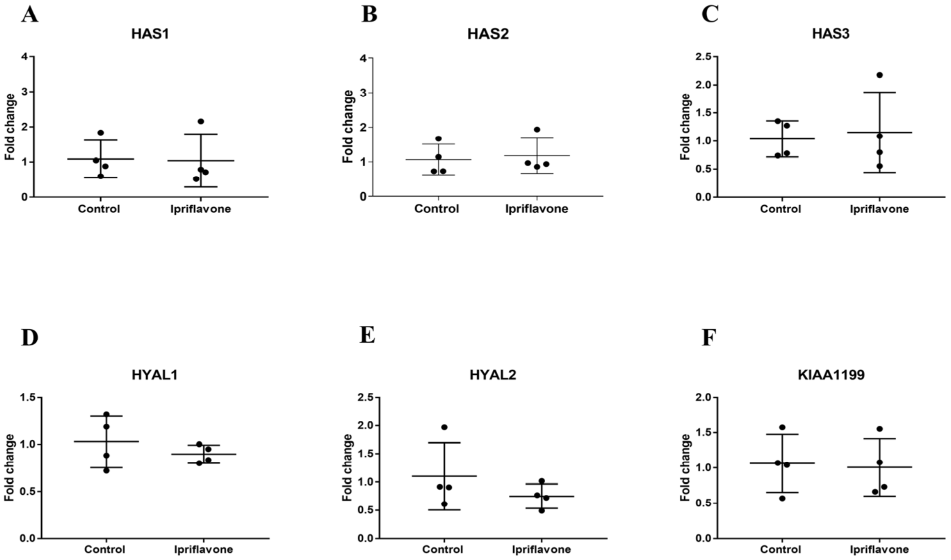

Figure S1. mRNA expression of ECM-related genes in mouse knee joint cartilage. mRNA expression of *HAS1* (A), *HAS2* (B), *HAS3* (C), *HYAL1* (D), *HYAL2* (E), and *KIAA1199* (F) in mouse knee joint cartilage from control group and ipriflavone group 10 weeks after the DMM surgery, which was determined by real-time PCR amplification (n = 4, respectively). The data presented are the average ± S.D. of the relative mRNA expression values normalized to GAPDH mRNA expression.
